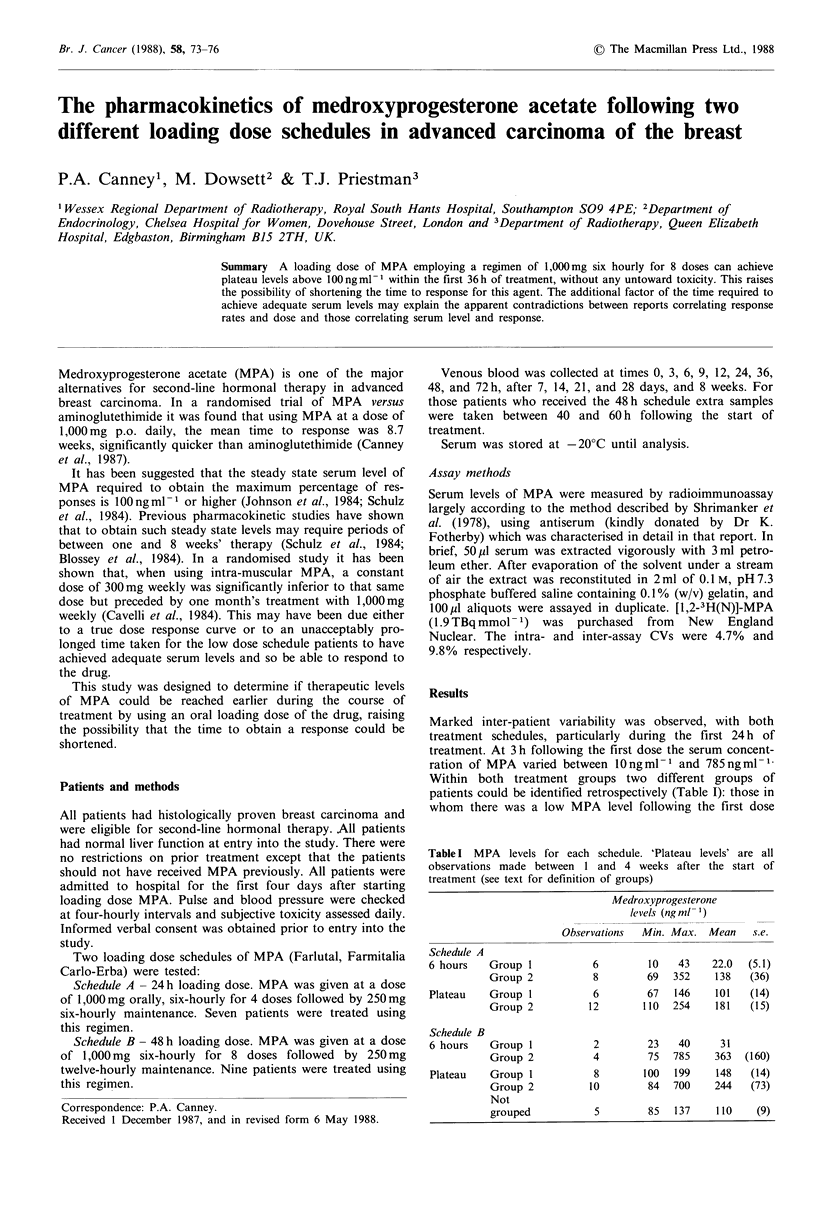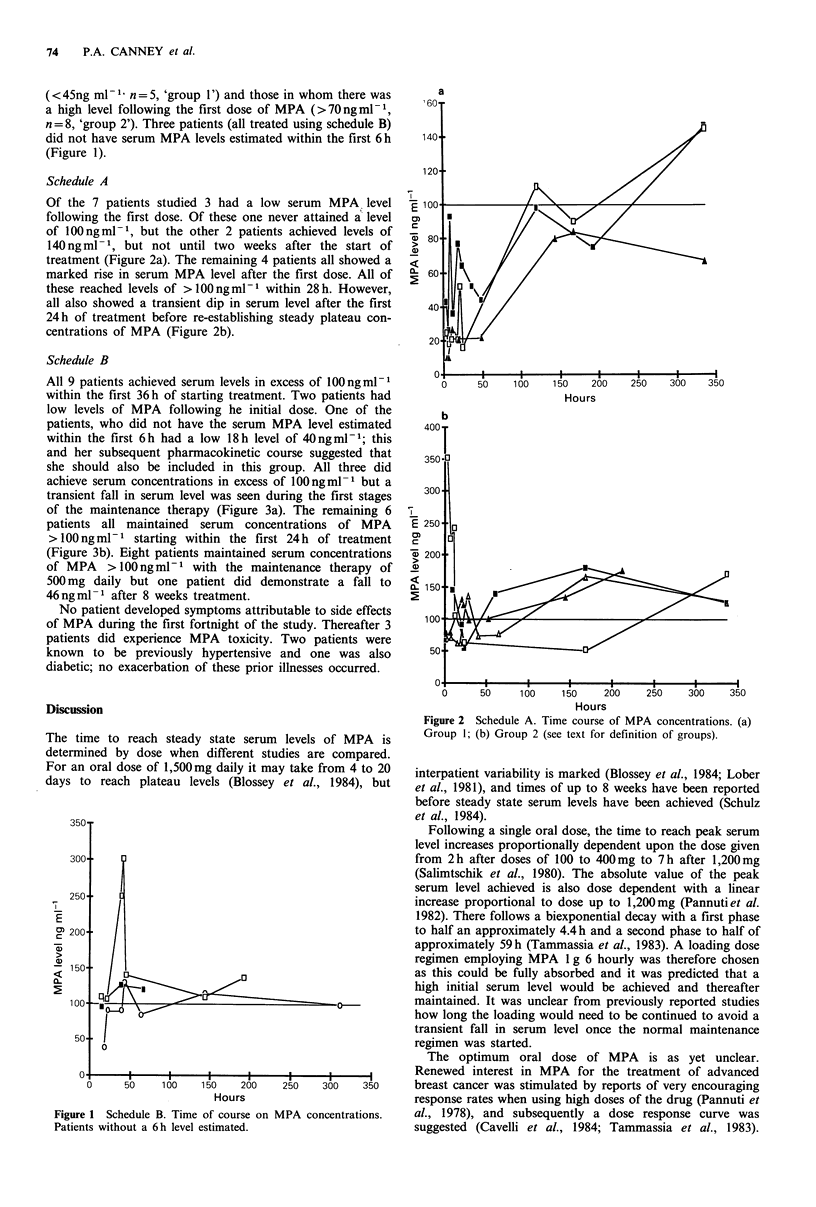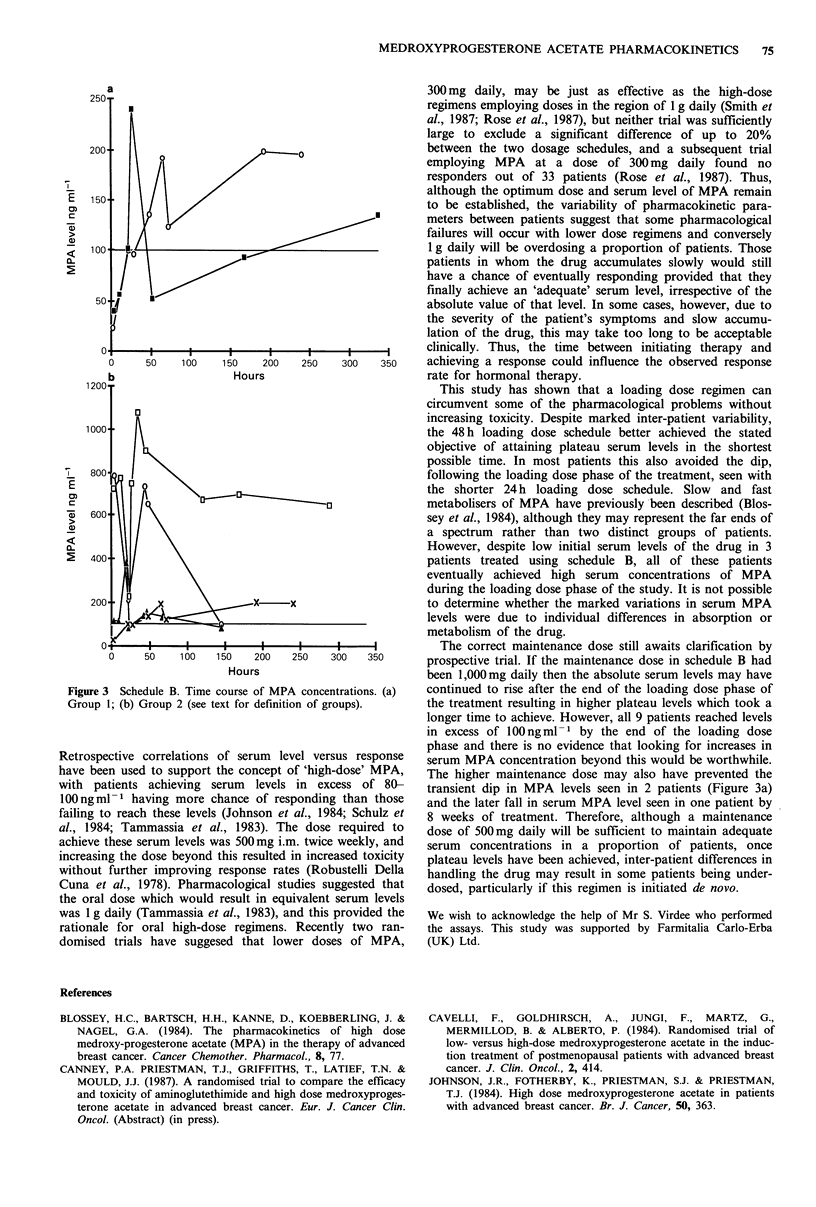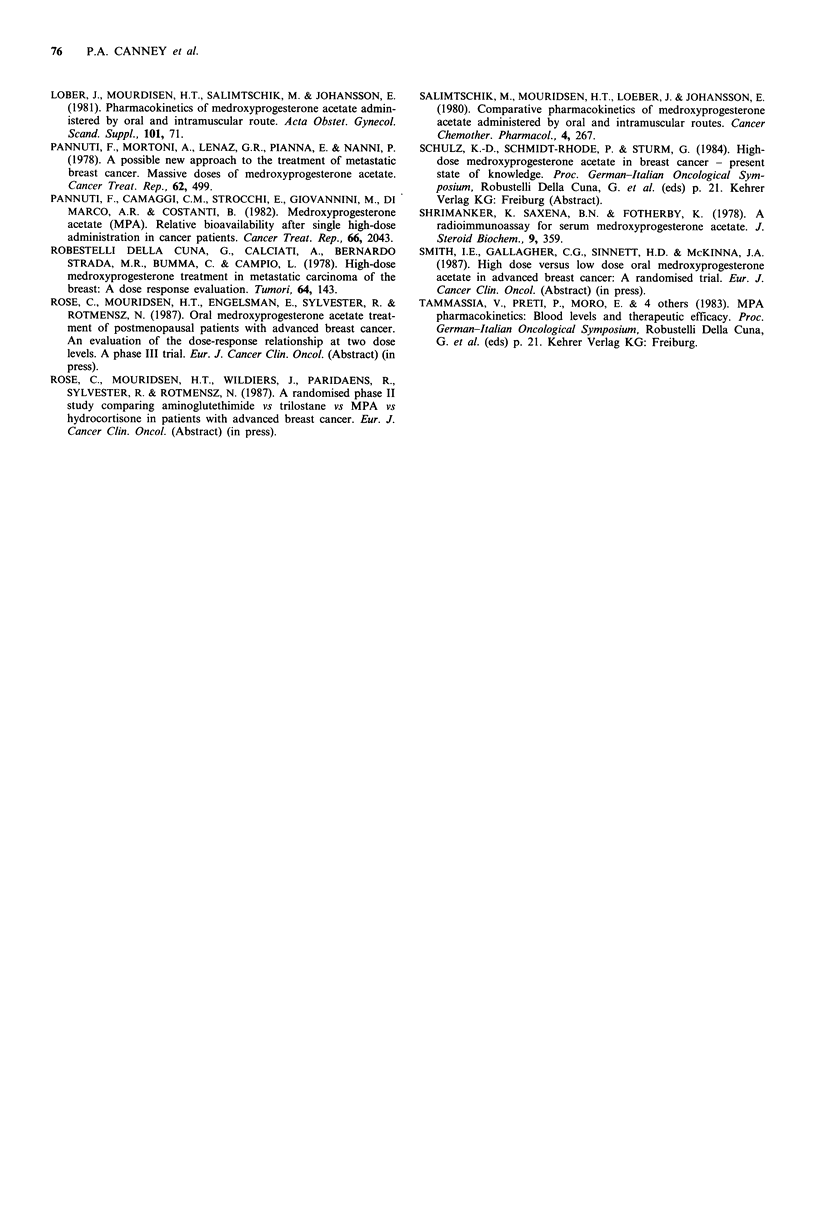# The pharmacokinetics of medroxyprogesterone acetate following two different loading dose schedules in advanced carcinoma of the breast.

**DOI:** 10.1038/bjc.1988.165

**Published:** 1988-07

**Authors:** P. A. Canney, M. Dowsett, T. J. Priestman

**Affiliations:** Wessex Regional Department of Radiotherapy, Royal South Hants Hospital, Southampton, UK.

## Abstract

A loading dose of MPA employing a regimen of 1,000 mg six hourly for 8 doses can achieve plateau levels above 100 ng ml-1 within the first 36 h of treatment, without any untoward toxicity. This raises the possibility of shortening the time to response for this agent. The additional factor of the time required to achieve adequate serum levels may explain the apparent contradictions between reports correlating response rates and dose and those correlating serum level and response.


Br. J. Cancer (1988), 58, 73–76 © The Macmillan Press Ltd., 1988

### The pharmacokinetics of medroxyprogesterone acetate following two different loading dose schedules in advanced carcinoma of the breast

P.A. Canney[1], M. Dowsett[2] & T.J. Priestman[3]

[1]*Wessex Regional Department of Radiotherapy, Royal South Hants Hospital, Southampton SO9 4PE;* [2]*Department of Endocrinology, Chelsea Hospital for Women, Dovehouse Street, London and* [3]*Department of Radiotherapy, Queen Elizabeth Hospital, Edgbaston, Birmingham B15 2TH, UK.*

**Summary** A loading dose of MPA employing a regimen of 1,000 mg six hourly for 8 doses can achieve plateau levels above 100 ng ml$^{-1}$ within the first 36 h of treatment, without any untoward toxicity. This raises the possibility of shortening the time to response for this agent. The additional factor of the time required to achieve adequate serum levels may explain the apparent contradictions between reports correlating response rates and dose and those correlating serum level and response.

Medroxyprogesterone acetate (MPA) is one of the major alternatives for second-line hormonal therapy in advanced breast carcinoma. In a randomised trial of MPA *versus* aminoglutethimide it was found that using MPA at a dose of 1,000 mg p.o. daily, the mean time to response was 8.7 weeks, significantly quicker than aminoglutethimide (Canney *et al.*, 1987).

It has been suggested that the steady state serum level of MPA required to obtain the maximum percentage of responses is 100 ng ml$^{-1}$ or higher (Johnson *et al.*, 1984; Schulz *et al.*, 1984). Previous pharmacokinetic studies have shown that to obtain such steady state levels may require periods of between one and 8 weeks' therapy (Schulz *et al.*, 1984; Blossey *et al.*, 1984). In a randomised study it has been shown that, when using intra-muscular MPA, a constant dose of 300 mg weekly was significantly inferior to that same dose but preceded by one month's treatment with 1,000 mg weekly (Cavelli *et al.*, 1984). This may have been due either to a true dose response curve or to an unacceptably prolonged time taken for the low dose schedule patients to have achieved adequate serum levels and so be able to respond to the drug.

This study was designed to determine if therapeutic levels of MPA could be reached earlier during the course of treatment by using an oral loading dose of the drug, raising the possibility that the time to obtain a response could be shortened.

### Patients and methods

All patients had histologically proven breast carcinoma and were eligible for second-line hormonal therapy. All patients had normal liver function at entry into the study. There were no restrictions on prior treatment except that the patients should not have received MPA previously. All patients were admitted to hospital for the first four days after starting loading dose MPA. Pulse and blood pressure were checked at four-hourly intervals and subjective toxicity assessed daily. Informed verbal consent was obtained prior to entry into the study.

Two loading dose schedules of MPA (Farlutal, Farmitalia Carlo-Erba) were tested:

*Schedule A* – 24 h loading dose. MPA was given at a dose of 1,000 mg orally, six-hourly for 4 doses followed by 250 mg six-hourly maintenance. Seven patients were treated using this regimen.

*Schedule B* – 48 h loading dose. MPA was given at a dose of 1,000 mg six-hourly for 8 doses followed by 250 mg twelve-hourly maintenance. Nine patients were treated using this regimen.

Correspondence: P.A. Canney.
Received 1 December 1987, and in revised form 6 May 1988.

Venous blood was collected at times 0, 3, 6, 9, 12, 24, 36, 48, and 72 h, after 7, 14, 21, and 28 days, and 8 weeks. For those patients who received the 48 h schedule extra samples were taken between 40 and 60 h following the start of treatment.

Serum was stored at −20°C until analysis.

### *Assay methods*

Serum levels of MPA were measured by radioimmunoassay largely according to the method described by Shrimanker *et al.* (1978), using antiserum (kindly donated by Dr K. Fotherby) which was characterised in detail in that report. In brief, 50 µl serum was extracted vigorously with 3 ml petroleum ether. After evaporation of the solvent under a stream of air the extract was reconstituted in 2 ml of 0.1 M, pH 7.3 phosphate buffered saline containing 0.1% (w/v) gelatin, and 100 µl aliquots were assayed in duplicate. [1,2-$^3$H(N)]-MPA (1.9 TBq mmol$^{-1}$) was purchased from New England Nuclear. The intra- and inter-assay CVs were 4.7% and 9.8% respectively.

### Results

Marked inter-patient variability was observed, with both treatment schedules, particularly during the first 24 h of treatment. At 3 h following the first dose the serum concentration of MPA varied between 10 ng ml$^{-1}$ and 785 ng ml$^{-1}$. Within both treatment groups two different groups of patients could be identified retrospectively (Table I): those in whom there was a low MPA level following the first dose

**Table I** MPA levels for each schedule. 'Plateau levels' are all observations made between 1 and 4 weeks after the start of treatment (see text for definition of groups)

| | | | *Medroxyprogesterone levels (ng ml$^{-1}$)* | | | |
|---|---|---|---|---|---|---|
| | | *Observations* | *Min.* | *Max.* | *Mean* | *s.e.* |
| *Schedule A* | | | | | | |
| 6 hours | Group 1 | 6 | 10 | 43 | 22.0 | (5.1) |
| | Group 2 | 8 | 69 | 352 | 138 | (36) |
| Plateau | Group 1 | 6 | 67 | 146 | 101 | (14) |
| | Group 2 | 12 | 110 | 254 | 181 | (15) |
| *Schedule B* | | | | | | |
| 6 hours | Group 1 | 2 | 23 | 40 | 31 | |
| | Group 2 | 4 | 75 | 785 | 363 | (160) |
| Plateau | Group 1 | 8 | 100 | 199 | 148 | (14) |
| | Group 2 | 10 | 84 | 700 | 244 | (73) |
| | Not grouped | 5 | 85 | 137 | 110 | (9) |

(<45ng ml$^{-1}$, $n=5$, 'group 1') and those in whom there was a high level following the first dose of MPA (>70 ng ml$^{-1}$, $n=8$, 'group 2'). Three patients (all treated using schedule B) did not have serum MPA levels estimated within the first 6 h (Figure 1).

*Schedule A*

Of the 7 patients studied 3 had a low serum MPA level following the first dose. Of these one never attained a level of 100 ng ml$^{-1}$, but the other 2 patients achieved levels of 140 ng ml$^{-1}$, but not until two weeks after the start of treatment (Figure 2a). The remaining 4 patients all showed a marked rise in serum MPA level after the first dose. All of these reached levels of >100 ng ml$^{-1}$ within 28 h. However, all also showed a transient dip in serum level after the first 24 h of treatment before re-establishing steady plateau concentrations of MPA (Figure 2b).

*Schedule B*

All 9 patients achieved serum levels in excess of 100 ng ml$^{-1}$ within the first 36 h of starting treatment. Two patients had low levels of MPA following he initial dose. One of the patients, who did not have the serum MPA level estimated within the first 6 h had a low 18 h level of 40 ng ml$^{-1}$; this and her subsequent pharmacokinetic course suggested that she should also be included in this group. All three did achieve serum concentrations in excess of 100 ng ml$^{-1}$ but a transient fall in serum level was seen during the first stages of the maintenance therapy (Figure 3a). The remaining 6 patients all maintained serum concentrations of MPA >100 ng ml$^{-1}$ starting within the first 24 h of treatment (Figure 3b). Eight patients maintained serum concentrations of MPA >100 ng ml$^{-1}$ with the maintenance therapy of 500 mg daily but one patient did demonstrate a fall to 46 ng ml$^{-1}$ after 8 weeks treatment.

No patient developed symptoms attributable to side effects of MPA during the first fortnight of the study. Thereafter 3 patients did experience MPA toxicity. Two patients were known to be previously hypertensive and one was also diabetic; no exacerbation of these prior illnesses occurred.

### Discussion

The time to reach steady state serum levels of MPA is determined by dose when different studies are compared. For an oral dose of 1,500 mg daily it may take from 4 to 20 days to reach plateau levels (Blossey *et al.*, 1984), but

**Figure 1** Schedule B. Time of course on MPA concentrations. Patients without a 6 h level estimated.

**Figure 2** Schedule A. Time course of MPA concentrations. (a) Group 1; (b) Group 2 (see text for definition of groups).

interpatient variability is marked (Blossey *et al.*, 1984; Lober *et al.*, 1981), and times of up to 8 weeks have been reported before steady state serum levels have been achieved (Schulz *et al.*, 1984).

Following a single oral dose, the time to reach peak serum level increases proportionally dependent upon the dose given from 2 h after doses of 100 to 400 mg to 7 h after 1,200 mg (Salimtschik *et al.*, 1980). The absolute value of the peak serum level achieved is also dose dependent with a linear increase proportional to dose up to 1,200 mg (Pannuti *et al.* 1982). There follows a biexponential decay with a first phase to half an approximately 4.4 h and a second phase to half of approximately 59 h (Tammassia *et al.*, 1983). A loading dose regimen employing MPA 1 g 6 hourly was therefore chosen as this could be fully absorbed and it was predicted that a high initial serum level would be achieved and thereafter maintained. It was unclear from previously reported studies how long the loading would need to be continued to avoid a transient fall in serum level once the normal maintenance regimen was started.

The optimum oral dose of MPA is as yet unclear. Renewed interest in MPA for the treatment of advanced breast cancer was stimulated by reports of very encouraging response rates when using high doses of the drug (Pannuti *et al.*, 1978), and subsequently a dose response curve was suggested (Cavelli *et al.*, 1984; Tammassia *et al.*, 1983).

**Figure 3** Schedule B. Time course of MPA concentrations. (a) Group 1; (b) Group 2 (see text for definition of groups).

Retrospective correlations of serum level versus response have been used to support the concept of 'high-dose' MPA, with patients achieving serum levels in excess of 80–100 ng ml$^{-1}$ having more chance of responding than those failing to reach these levels (Johnson *et al.*, 1984; Schulz *et al.*, 1984; Tammassia *et al.*, 1983). The dose required to achieve these serum levels was 500 mg i.m. twice weekly, and increasing the dose beyond this resulted in increased toxicity without further improving response rates (Robustelli Della Cuna *et al.*, 1978). Pharmacological studies suggested that the oral dose which would result in equivalent serum levels was 1 g daily (Tammassia *et al.*, 1983), and this provided the rationale for oral high-dose regimens. Recently two randomised trials have suggesed that lower doses of MPA, 300 mg daily, may be just as effective as the high-dose regimens employing doses in the region of 1 g daily (Smith *et al.*, 1987; Rose *et al.*, 1987), but neither trial was sufficiently large to exclude a significant difference of up to 20% between the two dosage schedules, and a subsequent trial employing MPA at a dose of 300 mg daily found no responders out of 33 patients (Rose *et al.*, 1987). Thus, although the optimum dose and serum level of MPA remain to be established, the variability of pharmacokinetic parameters between patients suggest that some pharmacological failures will occur with lower dose regimens and conversely 1 g daily will be overdosing a proportion of patients. Those patients in whom the drug accumulates slowly would still have a chance of eventually responding provided that they finally achieve an 'adequate' serum level, irrespective of the absolute value of that level. In some cases, however, due to the severity of the patient's symptoms and slow accumulation of the drug, this may take too long to be acceptable clinically. Thus, the time between initiating therapy and achieving a response could influence the observed response rate for hormonal therapy.

This study has shown that a loading dose regimen can circumvent some of the pharmacological problems without increasing toxicity. Despite marked inter-patient variability, the 48 h loading dose schedule better achieved the stated objective of attaining plateau serum levels in the shortest possible time. In most patients this also avoided the dip, following the loading dose phase of the treatment, seen with the shorter 24 h loading dose schedule. Slow and fast metabolisers of MPA have previously been described (Blossey *et al.*, 1984), although they may represent the far ends of a spectrum rather than two distinct groups of patients. However, despite low initial serum levels of the drug in 3 patients treated using schedule B, all of these patients eventually achieved high serum concentrations of MPA during the loading dose phase of the study. It is not possible to determine whether the marked variations in serum MPA levels were due to individual differences in absorption or metabolism of the drug.

The correct maintenance dose still awaits clarification by prospective trial. If the maintenance dose in schedule B had been 1,000 mg daily then the absolute serum levels may have continued to rise after the end of the loading dose phase of the treatment resulting in higher plateau levels which took a longer time to achieve. However, all 9 patients reached levels in excess of 100 ng ml$^{-1}$ by the end of the loading dose phase and there is no evidence that looking for increases in serum MPA concentration beyond this would be worthwhile. The higher maintenance dose may also have prevented the transient dip in MPA levels seen in 2 patients (Figure 3a) and the later fall in serum MPA level seen in one patient by 8 weeks of treatment. Therefore, although a maintenance dose of 500 mg daily will be sufficient to maintain adequate serum concentrations in a proportion of patients, once plateau levels have been achieved, inter-patient differences in handling the drug may result in some patients being underdosed, particularly if this regimen is initiated *de novo*.

We wish to acknowledge the help of Mr S. Virdee who performed the assays. This study was supported by Farmitalia Carlo-Erba (UK) Ltd.

### References

BLOSSEY, H.C., BARTSCH, H.H., KANNE, D., KOEBBERLING, J. & NAGEL, G.A. (1984). The pharmacokinetics of high dose medroxy-progesterone acetate (MPA) in the therapy of advanced breast cancer. *Cancer Chemother. Pharmacol.*, **8**, 77.

CANNEY, P.A. PRIESTMAN, T.J., GRIFFITHS, T., LATIEF, T.N. & MOULD, J.J. (1987). A randomised trial to compare the efficacy and toxicity of aminoglutethimide and high dose medroxyprogesterone acetate in advanced breast cancer. *Eur. J. Cancer Clin. Oncol.* (Abstract) (in press).

CAVELLI, F., GOLDHIRSCH, A., JUNGI, F., MARTZ, G., MERMILLOD, B. & ALBERTO, P. (1984). Randomised trial of low- versus high-dose medroxyprogesterone acetate in the induction treatment of postmenopausal patients with advanced breast cancer. *J. Clin. Oncol.*, **2**, 414.

JOHNSON, J.R., FOTHERBY, K., PRIESTMAN, S.J. & PRIESTMAN, T.J. (1984). High dose medroxyprogesterone acetate in patients with advanced breast cancer. *Br. J. Cancer*, **50**, 363.

LOBER, J., MOURDISEN, H.T., SALIMTSCHIK, M. & JOHANSSON, E. (1981). Pharmacokinetics of medroxyprogesterone acetate administered by oral and intramuscular route. *Acta Obstet. Gynecol. Scand. Suppl.*, **101**, 71.

PANNUTI, F., MORTONI, A., LENAZ, G.R., PIANNA, E. & NANNI, P. (1978). A possible new approach to the treatment of metastatic breast cancer. Massive doses of medroxyprogesterone acetate. *Cancer Treat. Rep.*, **62**, 499.

PANNUTI, F., CAMAGGI, C.M., STROCCHI, E., GIOVANNINI, M., DI MARCO, A.R. & COSTANTI, B. (1982). Medroxyprogesterone acetate (MPA). Relative bioavailability after single high-dose administration in cancer patients. *Cancer Treat. Rep.*, **66**, 2043.

ROBESTELLI DELLA CUNA, G., CALCIATI, A., BERNARDO STRADA, M.R., BUMMA, C. & CAMPIO, L. (1978). High-dose medroxyprogesterone treatment in metastatic carcinoma of the breast: A dose response evaluation. *Tumori*, **64**, 143.

ROSE, C., MOURIDSEN, H.T., ENGELSMAN, E., SYLVESTER, R. & ROTMENSZ, N. (1987). Oral medroxyprogesterone acetate treatment of postmenopausal patients with advanced breast cancer. An evaluation of the dose-response relationship at two dose levels. A phase III trial. *Eur. J. Cancer Clin. Oncol.* (Abstract) (in press).

ROSE, C., MOURIDSEN, H.T., WILDIERS, J., PARIDAENS, R., SYLVESTER, R. & ROTMENSZ, N. (1987). A randomised phase II study comparing aminoglutethimide *vs* trilostane *vs* MPA *vs* hydrocortisone in patients with advanced breast cancer. *Eur. J. Cancer Clin. Oncol.* (Abstract) (in press).

SALIMTSCHIK, M., MOURIDSEN, H.T., LOEBER, J. & JOHANSSON, E. (1980). Comparative pharmacokinetics of medroxyprogesterone acetate administered by oral and intramuscular routes. *Cancer Chemother. Pharmacol.*, **4**, 267.

SCHULZ, K.-D., SCHMIDT-RHODE, P. & STURM, G. (1984). High-dose medroxyprogesterone acetate in breast cancer – present state of knowledge. *Proc. German–Italian Oncological Symposium*, Robustelli Della Cuna, G. *et al.* (eds) p. 21. Kehrer Verlag KG: Freiburg (Abstract).

SHRIMANKER, K. SAXENA, B.N. & FOTHERBY, K. (1978). A radioimmunoassay for serum medroxyprogesterone acetate. *J. Steroid Biochem.*, **9**, 359.

SMITH, I.E., GALLAGHER, C.G., SINNETT, H.D. & McKINNA, J.A. (1987). High dose versus low dose oral medroxyprogesterone acetate in advanced breast cancer: A randomised trial. *Eur. J. Cancer Clin. Oncol.* (Abstract) (in press).

TAMMASSIA, V., PRETI, P., MORO, E. & 4 others (1983). MPA pharmacokinetics: Blood levels and therapeutic efficacy. *Proc. German–Italian Oncological Symposium*, Robustelli Della Cuna, G. *et al.* (eds) p. 21. Kehrer Verlag KG: Freiburg.